# Central statistical monitoring in clinical trials

**DOI:** 10.1186/1745-6215-12-S1-A55

**Published:** 2011-12-13

**Authors:** Amy A Kirkwood, Allan Hackshaw

**Affiliations:** 1 CRUK and UCL Cancer Trials Centre, London, W1T 4TJ, UK

## Background

On-site monitoring is a common but time-consuming and expensive activity, with little evidence that it is worthwhile. Centralised statistical monitoring (CSM) is a much cheaper alternative, where data checks are performed by the co-ordinating centre, reducing the need to visit every site. Although some publications have outlined possible methods, few have applied them to data from real clinical trials.

## Methods

R-programs were developed to check data at either the patient or site level, for fraud or data errors. These included finding anomalous data patterns, digit preference, rounding, incorrect dates (eg weekends/holidays), values of variables too close or too far from the means, odd correlation structures and extreme values or variances. We applied these to 3 trials: (i) where data had already been checked, (ii) an ongoing trial where our findings could be checked in real-time, and (iii) where data errors and fake patients were created.

## Findings

**Figure 1:**
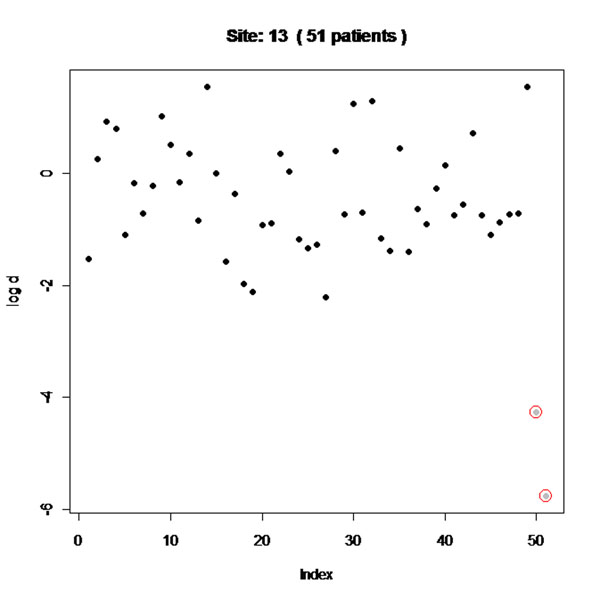
Patient level data checks. Output for one site, in which data were faked for 2 patients (shown in grey) by creating values for several variables that were close to the mean of all patients (which is more likely to occur if data were to be faked). Patients with values which lie too close to the multivariate mean are shown away from the others and were picked up (and circled in red) by the program.

**Figure 2:**
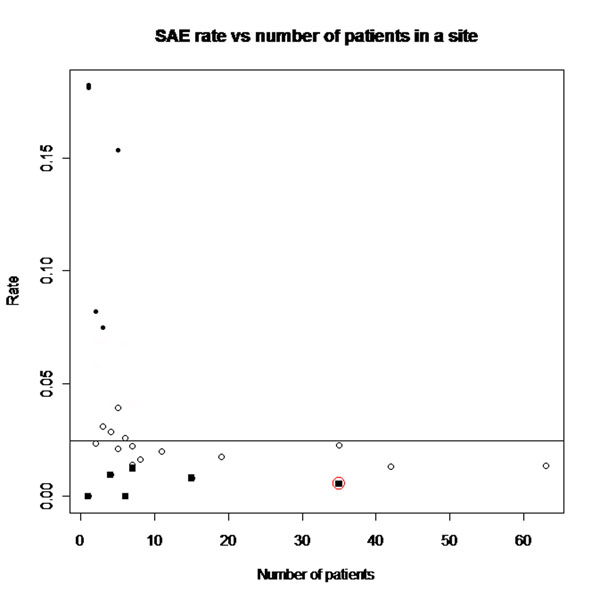
Site level data checks. The y-axis represents the SAE rate per site, allowing for time in the trial by patients. The lowest 10% of SAE rates are shown as black squares. The circled observation is for a site where data were faked so that the site had too few SAEs, compared to the average for all sites (horizontal line). Sites in the bottom right hand corner have lower than expected SAE rates but relatively large numbers of patients, so could have on-site monitoring checks.

The programs were designed to be run automatically and produce simple tables or figures. Few errors were detected in the trial where data had already been checked (as expected). Most data errors were found in the two other trials. The programs were able to detect data errors, as well as fabricated patients that we generated to have values that were too close to the multivariate mean (fig. 1). They also detected centres that had too few or too many serious adverse events (fig. 2). It might be difficult to reliably apply some of the programs to centres with few patients. Several patients that were fabricated were not detected because the data did not follow the assumptions used by the R-programs, or the number of fabricated patients within a centre was too small. Examples of the different output produced, including easy-to-read diagrams and how they are interpreted, could be shown and discussed, along with their strengths and limitations.

## Conclusions

CSM appears to be a cost-effective and worthwhile alternative to on-site monitoring. It can identify incorrect patient data, or centre where the data considered together is too different to all other sites and therefore should be reviewed. However, more research is needed to identify which situations CSM does not work well in.

